# Predicting acute respiratory distress syndrome in influenza pneumonia patients using delta mean platelet volume

**DOI:** 10.1186/s12890-021-01763-5

**Published:** 2021-12-07

**Authors:** Teeraphat Reangvilaikul, Piyaphat Udompongpaiboon, Veerapong Vattanavanit

**Affiliations:** 1 Faculty of Medicine, Prince of Songkla University, 15 Kanjanavanich Road, Hat Yai, Songkhla, 90110 Thailand; 2 Critical Care Medicine Unit, Division of Internal Medicine, Faculty of Medicine, Prince of Songkla University, 15 Kanjanavanich Road, Hat Yai, Songkhla, 90110 Thailand

**Keywords:** Influenza, Pneumonia, ARDS, Mean platelet volume

## Abstract

**Background:**

Patients with influenza pneumonia are at high risk of rapid progression to acute respiratory distress syndrome (ARDS). Mean platelet volume (MPV), which reflects platelet size, is considered to be a crucial inflammatory marker. The study aim was to investigate the role of delta mean platelet volume (delta MPV) in predicting ARDS in patients with influenza pneumonia.

**Methods:**

This retrospective study was conducted in a tertiary care centre in southern Thailand. Adult patients diagnosed with influenza pneumonia were enrolled from January 2015 to December 2020. Demographic data, laboratory investigations including delta MPV (MPV on day 2 minus MPV on day 1), management records, and clinical outcomes were collected for analysis. The study population was divided into two groups according to the development of ARDS.

**Results:**

During the study, 1240 patients with laboratory-confirmed influenza were screened and 212 pneumonia patients were enrolled. Fifty-six patients (26.4%) met the diagnostic criteria for ARDS during hospitalization. Delta MPV was significantly higher in the ARDS group compared to that in the non-ARDS group (1.0 fL vs 0.2 fL, p < 0.001). Multivariable logistic regression revealed that delta MPV is an independent predictor of ARDS (OR 17.37; 95% CI 6.5–46.4; p < 0.001). Receiver operating characteristic curve analysis indicated a cut-off value of 0.7 fL for delta MPV (sensitivity 80.36%, specificity 80.77%) to predict ARDS in patients with influenza pneumonia.

**Conclusions:**

Delta MPV strongly predicts ARDS in influenza pneumonia patients. Implementation of delta MPV may be useful in identifying at-risk patients who will require intensive care and ARDS prevention.

**Supplementary Information:**

The online version contains supplementary material available at 10.1186/s12890-021-01763-5.

## Background

The influenza virus is a leading cause of viral pneumonia worldwide. Data from the 2009 H1N1 pandemic show that 49–72% of patients with pneumonia developed acute respiratory distress syndrome (ARDS), leading to high morbidity and mortality rates [1, 2]. Early recognition and risk stratification are necessary to improve outcomes of influenza pneumonia patients.

There is a growing body of clinical evidence suggesting that platelets play an important role in immune and inflammatory responses. Mean platelet volume (MPV) is an indicator of platelet size and activity, which is simply measured using automated haematology analysers. An increased platelet size and volume reflects thrombosis and inflammatory processes [3]. A single measurement of elevated MPV has been reported to be associated with increased morbidity in various critical conditions [4, 5]. Moreover, changes in MPV have been reported as a dynamic parameter that is associated with poor outcome in critically ill patients and pneumonia patients [6, 7]. However, the MPV changes or delta MPV discussed in previous studies have different definitions, both in terms of timing and cut off [6, 7]. To our knowledge, there is little data on delta MPV in prediction of ARDS in patients with influenza pneumonia.

The aim of our study was to investigate the predictive value of MPV change or delta MPV in the development of ARDS in patients with influenza pneumonia.

## Methods

### Study population and design

This retrospective study was conducted using a health information system database of patients admitted to wards or the medical intensive care unit (ICU) of Songklanagarind Hospital (a university-affiliated, 800-bed tertiary hospital in southern Thailand) between January 2015 and December 2020.

Patients were included if they had been admitted with pneumonia and had influenza infection. Patients were excluded from this study if they were younger than 18 years old, had initial presentation of ARDS, or inadequate complete blood cell count data. The protocol of this study was approved by the ethics committee of the Faculty of Medicine, Prince of Songkla University (EC number: 63-482-14-1). The requirement for informed consent was waived due to the retrospective nature of the study.

### Data collection

The study participants’ epidemiologic and clinical data were obtained from the electronic medical record. The data included age, sex, height, body weight, comorbid conditions, illness severity scores, laboratory values, respiratory intervention and clinical outcomes. Complete blood cell counts including MPV values were determined using an automated haematology analyser (XT-1800i, Sysmex Corporation, Japan).

### Definitions

Influenza infection was confirmed by a positive result from one of the following tests: rapid antigen test, or nucleic reverse transcriptase polymerase chain reaction (RT-PCR) from nasopharyngeal swab, throat swab, sputum or bronchoalveolar lavage.

Pneumonia was defined as the presence of at least one of the following: fever (body temperature > 38.2 °C) or hypothermia (temperature < 35.0 °C), new cough with or without sputum production, dyspnoea or altered breathing sound on auscultation, or presence of new chest radiographic infiltrates [8, 9]. ARDS was diagnosed according to the Berlin definition: acute onset within 1 week, bilateral lung opacities, no evidence of cardiac failure-related hydrostatic oedema by echocardiography, and PaO_2_/FiO_2_ ratio < 300 mm Hg with positive end-expiratory pressure (PEEP) ≥ 5 cm H_2_O [10].

Illness severity was determined using acute physiology and chronic health evaluation II (APACHE II) [11], sequential organ failure assessment (SOFA) score [12], national early warning score (NEWS) [13], CURB-65 (confusion, urea > 7 mmol/L, respiratory rate ≥ 30/min, blood pressure [systolic < 90 mm Hg or diastolic ≤ 60 mm Hg], and age ≥ 65 years) pneumonia severity score [14], and pneumonia severity index (PSI) [15]. All components of the variables of APACHE II, SOFA, NEWS, CURB-65 and PSI were recorded, capturing the highest and lowest value during the first 24 h of hospital admission (Additional file 1).

Day 1 was defined as the interval from admission to 5:00 am the next day; all other days were calendar days from 5:00 am to 4.59 am.

Delta MPV was defined as MPV on day 2 minus MPV on day 1. Two sets of CBC were selected to determine delta MPV, the first one at the time of admission, and the second one on the next calendar day when blood samples are routinely taken at 6 am. MPV/platelet count ratio (MPR) was defined as MPV (fL)/platelet count (^10^9^/L) × 100%.

### Statistical analysis

The sample size was determined using sample size estimation for diagnostic test studies [16]. Estimating that 30% of influenza pneumonia patients have ARDS, we calculated that a sample size of 205 would provide 95% confidence with 80% power to detect a difference of 10% from the presumptive value of 80% for sensitivity.

The data were tested for normality using the Shapiro–Wilk test. Categorical data are expressed as percentages. Continuous data are shown as mean ± standard deviation or median with minimum and maximum interquartile range (IQR), depending on the distribution of the data. Continuous variables and proportions were compared between groups using Student’s *t*-test or the Mann–Whitney *U* test and chi-square tests, respectively.

We assessed the association between clinical characteristics and ARDS using multivariable logistic regression analysis. Variables that were associated with ARDS (*P* < 0.2) were introduced into a multiple logistic regression model after testing for association. Collinearity between variables was excluded before modelling. Odds ratios (ORs) and their 95% confidence intervals (CIs) were used to identify the significant independent factors influencing ARDS.

Afterwards, a receiver operating characteristic (ROC) curve and a calculated corresponding area under the ROC curve (AUROC) of delta MPV and selected variables were constructed. The Youden index was introduced to select the best cut-off values of delta MPV with the best sensitivity, specificity, positive likelihood ratio (LR +), and negative likelihood ratio (LR-) for predicting ARDS. Two-tailed values of *P* < 0.05 were deemed statistically significant. All statistical analyses were computed using STATA version 16 (StataCorp, College station TX, USA).

## Results

### Patient characteristics

**Fig. 1:**
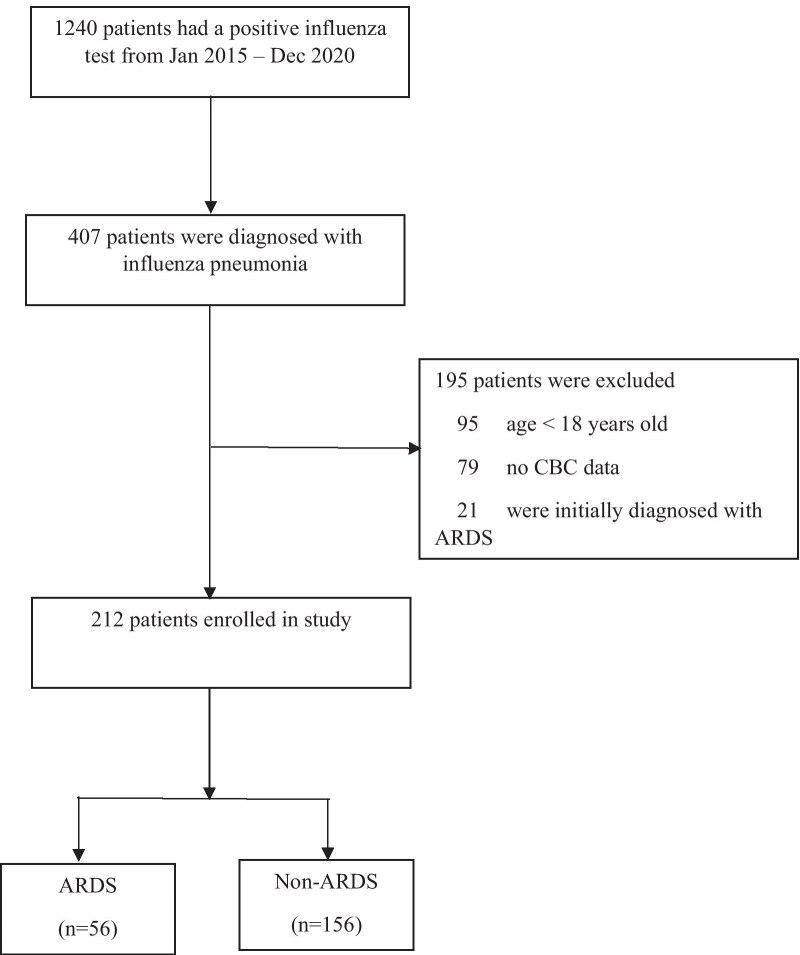
Study flow diagram. ARDS, acute respiratory distress syndrome; CBC, complete blood count

Of 1240 patients with a positive test for influenza between January 2015 and December 2020, 212 were enrolled in the study (Fig. 1). The median (interquartile range, IQR) patient age was 71 (59–79) years, and 110 (51.9%) were female. The median SOFA score was 2 (1–3) and the median APACHE II score was 12 (8–15). The most common type of influenza was influenza A (80.7%). Overall, in-hospital mortality was 9%.

### Clinical characteristics between ARDS and non-ARDS groups

**Fig. 2:**
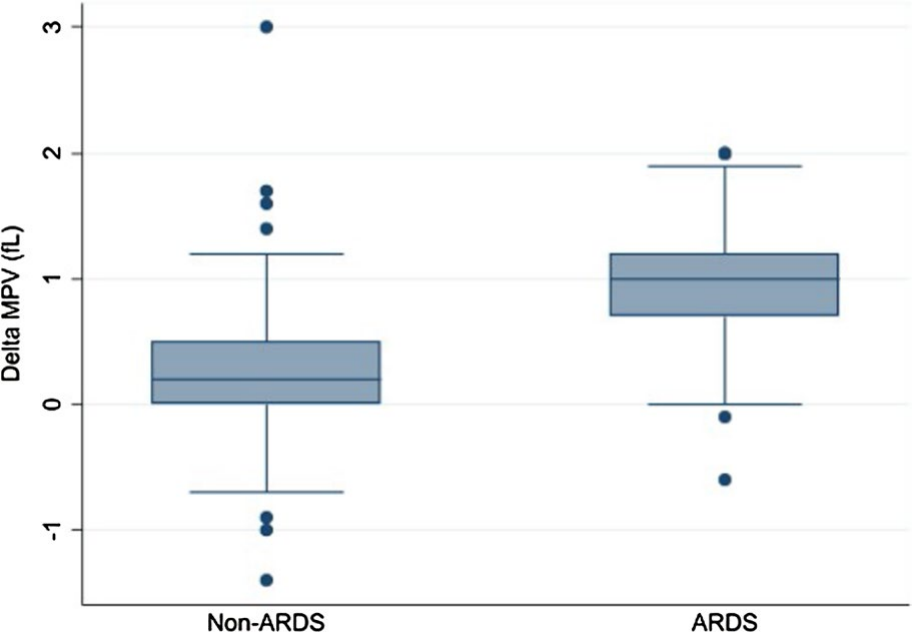
Comparison of delta MPV in ARDS and non-ARDS groups. ARDS, acute respiratory distress syndrome; MPV, mean platelet volume

The baseline demographic, clinical, and biochemical data of each group, stratified according to ARDS, are presented in Table 1. Of 212 pneumonia patients, 56 (26.4%) patients were diagnosed with ARDS. The median number of days from hospital admission to develop ARDS was 3 (1–6) days (Additional file 1). Patients with ARDS had significantly higher severity scores, including SOFA score [3 (2–4) vs 2 (1–3), p < 0.001], APACHE II score [14 (12–18.8) vs 12 (7–13), p < 0.001], CURB-65 [2 (2–3) vs 2 (1–2), p < 0.001] and PSI [99.5 (87.2–108.8) vs 88 (69–103.8), p = 0.006], than the non-ARDS group. White blood cell count [10.7 (6.9–13.8) vs 9.2 (6.3–12.7) × 10^3^/µL, p = 0.024], MPV on day 2 [11 (10.3–11.5) vs 10.3 (9.6–10.9) fL, p < 0.001], MPR on day 2 [6.4 (4.4–8.4) vs 4.8 (3.6–6.9), p = 0.002] and delta MPV [1 (0.7–1.2) vs 0.2 (0–0.5) fL, p < 0.001] were significantly higher in the ARDS group, whilst the percentage of lymphocytes was significantly lower in the ARDS group compared with the non-ARDS group [8.9 (5.9–14.4) vs 12 (7–19.1), p = 0.008]. Comparison of delta MPV between the two groups is shown in Fig. 2. The ARDS group had a longer length of hospital stay and higher mortality rate [21 (12–23.8) vs 7 (4–11) days, p < 0.001; 26.8% vs 2.6%, p < 0.001] (Table 1).

**Table 1:** Baseline characteristics of the patients according to diagnosis of ARDS

Variables	Total (n = 212)	Diagnosis		*p* value
		ARDS (n = 56)	Non-ARDS (n = 156)	
*Demographic data*				
Sex (female)	110 (51.9)	28 (50)	82 (52.6)	0.742
Age (years)	71 (59–79)	74 (64.25–83)	70 (58–78.75)	0.069
*Underlying diseases*				
Diabetes mellitus	57 (26.9)	16 (28.6)	41 (26.3)	0.740
Hypertension	79 (37.3)	25 (44.6)	54 (34.6)	0.183
History of malignancy	25 (11.8)	4 (7.1)	21 (13.5)	0.209
Immunosuppression	15 (7.1)	0	15 (9.6)	0.016
Chronic kidney disease	28 (13.2)	10 (17.9)	18 (11.5)	0.231
Coronary artery disease	20 (9.4)	6 (10.7)	14 (9)	0.702
Stroke	21 (9.9)	7 (12.5)	14 (9)	0.449
Cirrhosis	11 (5.2)	2 (3.6)	9 (5.8)	0.525
Chronic airway disease	35 (16.5)	7 (12.5)	28 (17.9)	0.346
Congestive heart failure				
*Clinical data*				
Charlson comorbidity index	2 (1–4)	2 (1–4)	2 (1–4.75)	0.297
SOFA	2 (1–3)	3 (2–4)	2 (1–3)	< 0.001
APACHE II	12 (8–15)	14 (12.0–18.75)	12 (7–13)	< 0.001
NEWS	6 (4–8)	7 (6–9)	5 (3–7)	< 0.001
CURB-65	2 (1–2)	2 (2–3)	2 (1–2)	< 0.001
PSI	92 (74–107)	99.5 (87.25–108.75)	88 (69–103.75)	0.006
*Influenza type*				
A	171 (80.7)	43 (76.8)	128 (82.1)	0.392
B	22 (19.8)	13 (23.2)	28 (17.9)	0.256
*Test positive*				
Rapid (IFA)	120 (56.6)	25 (44.6)	61 (39.1)	0.031
PCR	92 (43.4)	31 (55.4)	95 (60.9)	0.035
Haemoculture positive results	5/189	1/55	4/134	0.650
Sputum culture positive results	55/153	22/53	33/100	0.297
Interval between collection of CBC day 1 and day 2 (hours)	13 (7–17)	12 (7–16)	13 (7–18)	0.338
*Laboratory results*				
Hb level day 1 (g/dL)	11.9 (10.0–13.47)	12.35 (9.68–13.42)	11.75 (10.12–13.47)	0.478
WBC count day 1 (× 10^3^/µL)	9.66 (6.6–13.1)	10.74 (6.97–13.83)	9.2 (6.34–12.74)	0.024
PMN (%)	78 (69–85)	79.1 (69–84.6)	77.7 (68.4–85.15)	0.871
Lymphocytes (%)	10.85 (6.17–17.85)	8.9 (5.9–14.4)	12 (7–19.1)	0.008
Platelet count day 1 (× 10^3^/µL)	214 (161.2–280)	220.96 (152.25–289)	229.12 (162.25–280)	0.429
MPV day 1 (fL)	10 (9.4–10.6)	10.04 (9.45–10.4)	10.05 (9.4–10.6)	0.874
MPR day 1	4.64 (3.4–6.3)	5.12 (3.27–6.64)	4.5 (3.37–6.29)	0.512
MPV day 2 (fL)	10.5 (9.9–11.1)	11 (10.3–11.5)	10.3 (9.6–10.9)	< 0.001
MPR day 2	5.22 (3.8–7.2)	6.41 (4.45–8.36)	4.85 (3.63–6.99)	0.002
Delta MPV (fL)	0.4 (0.1–0.9)	1.0 (0.7–1.2)	0.2 (0–0.5)	< 0.001
*Outcomes*				
Invasive mechanical ventilation	99 (46.7)	54 (96.4)	45 (28.8)	< 0.001
Length of hospital stay (days)	9 (5–19)	21 (12–32.75)	7 (4–11)	< 0.001
In-hospital mortality	19 (9)	15 (26.8)	4 (2.6)	< 0.001

Data are presented as median (interquartile range) or n (%)

APACHE II, acute physiology and chronic health evaluation II; ARDS, acute respiratory distress syndrome; CURB-65, confusion, urea nitrogen, respiratory rate, blood pressure, 65 years of age and older; IFA, indirect fluorescent antibody; MPR, mean platelet count volume to platelet count ratio; MPV, mean platelet volume; NEWS, national early warning score; PCR, polymerase chain reaction; PMN, polymorphonuclear leukocytes; PSI, pneumonia severity index; SOFA, sequential organ failure assessment; WBC, white blood cells

### Prediction of ARDS

Multivariable logistic regression analysis revealed that delta MPV, APACHE II scores and CURB-65 are independent predictors of ARDS in patients with influenza pneumonia (Table 2).

**Table 2:** Predictors of ARDS

Characteristic	Crude OR (95% CI)	*p*	Adjusted OR (95% CI)	*P*
SOFA	1.402 (1.186–1.656)	< 0.001	1.023 (0.786–1.332)	0.865
APACHE II	1.158 (1.086–1.234)	< 0.001	1.137 (1.023–1.263)	0.017
NEWS	1.245 (1.117–1.388)	< 0.001	1.053 (0.895–1.238)	0.533
CURB-65	2.028 (1.365–3.012)	< 0.001	2.095 (1.159–3.787)	0.014
PSI	1.015 (1.004–1.026)	0.007	0.984 (0.963–1.004)	0.116
Delta MPV	14.476 (6.261–33.469)	< 0.001	17.374 (6.501–46.432)	< 0.001
MPR day 2	1.026 (0.986–1.068)	0.202	–	–

APACHE II, acute physiology and chronic health evaluation II; ARDS, acute respiratory distress syndrome; CURB-65, confusion, urea nitrogen, respiratory rate, blood pressure, 65 years of age and older; MPR, mean platelet count volume to platelet count ratio; MPV, mean platelet volume; NEWS, national early warning score; PSI, pneumonia severity index; SOFA, sequential organ failure assessment

**Fig. 3:**
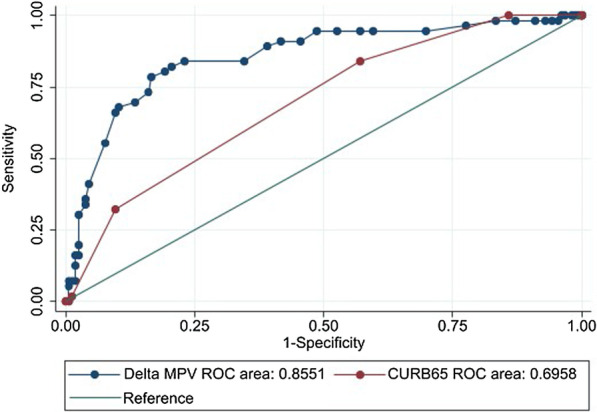
Receiver operating characteristic (ROC) curves demonstrating the predictive value of delta MPV and CURB-65 for ARDS. ARDS, acute respiratory distress syndrome; CURB-65, confusion, urea nitrogen, respiratory rate, blood pressure, 65 years of age and older; MPV, mean platelet volume

ROC curve analysis showed better performance of delta MPV than CURB-65 in predicting ARDS (AUROC 0.855 vs 0.696) (Fig. 3). A cut-off value of 0.7 fL for delta MPV (sensitivity 80.36%, specificity 80.77%, LR + 4.18, LR- 0.24) and ≥ 2 for CURB-65 (sensitivity 83.93%, specificity 42.95%, LR + 1.47, LR- 0.37) was used to predict ARDS in influenza pneumonia patients.

## Discussion

To our knowledge, this study is the first to investigate the association between ARDS and delta MPV in patients with influenza pneumonia. Our results indicate that delta MPV after hospital admission is an independent predictor of ARDS occurrence. In addition, delta MPV had greater predictive value than the well-known pneumonia severity score, CURB-65.

A recent study showed that changing values of MPV are associated with poor outcome in pneumonia. Lee et al. [6] reported that an increase in MPV on day 2, 3, 4 and discharge was associated with mortality in ICU patients with pneumonia. Gorelik et al. [17] revealed that a rise in mean platelet volume from admission to hospital discharge in pneumonia patients predicts the need for invasive mechanical ventilation and in-hospital mortality. However, using MPV at discharge may not provide advantages in ARDS prediction. According to our results, ARDS occurred on day 3 after admission on average. Useful predictor parameters should be obtained before this timepoint.

The single values of MPV on day 1 or day 2 did not differ significantly between the two groups. Our results support a previous meta-analysis that suggested that initial values of MPV may not be useful as a prognostic marker of mortality in critically ill patients [18].

In addition to delta MPV, MPR on day 1 was reported as a predictor of severe pneumonia in coronavirus disease 2019 (COVID-19) patients [19]. In our study, only MPR on day 2 was significantly higher in the ARDS group, but was not associated with ARDS in the univariable analysis. A recent study showed that delta MPV between the first and the third day of hospitalization predicts mortality in COVID-19 patients [20].

Delta MPV in our results was a better predictor of ARDS than other recognised pneumonia severity scores. This may be because these scores were developed from community-acquired pneumonia patients, mostly infected with bacteria, and were designed for mortality prediction [14, 15]. However, CURB-65 may be beneficial in ARDS prediction.

Our data provided some useful epidemiological information. Pneumonia occurred in approximately 32.8% of patients with a positive influenza test, and 26.4% of pneumonia patients developed ARDS. We found that influenza A was more common than influenza B, which is comparable to a previous study performed at the largest hospital in Thailand [21]. Influenza A causes more severe disease than influenza B [21, 22]. White blood cell counts in the ARDS group were significantly higher than in the non-ARDS group. Patients in the ARDS group had lower lymphocyte counts compared to the non-ARDS group. Our results are compatible with those from a study by Chen et al. [23] conducted during the severe acute respiratory syndrome (SARS) epidemic, which showed that ARDS patients tended to present with more severe lymphopenia and leukocytosis.

MPV reflects the increasing production of platelets, which are involved in the complex pathogenesis of ARDS. This involves dysregulated coagulation and an excessive inflammatory response mediated by cytokines released from platelet-activated ARDS processes [24]. Antiplatelet therapy may be beneficial in halting the ARDS process [25].

Our results were used to develop the ARDS prediction tools for patients with influenza pneumonia. If patients show delta MPV > 0.7 or CURB-65 ≥ 2, the physician should pay careful attention to ARDS prevention. Our ventilator parameter data revealed an excessive tidal volume as an initial setting, and close monitoring was recommended for preventing ARDS [26].

This study has some limitations. First, this is a single-centre study, leading to limitations in the generalizability of the results. Second, due to the retrospective design, selection bias or missing data may have distorted the results. Third, we did not evaluate all possible causes of a rise in MPV, such as renal dysfunction [27]. Further prospective studies on a larger patient population are required to establish the exact role of delta MPV in patients with influenza pneumonia, either as a predictor of ARDS or for the evaluation of response to treatment.

## Conclusions

Delta MPV strongly predicted ARDS in patients with influenza pneumonia. Patients with delta MPV > 0.7 should receive ARDS prevention interventions.

## Supplementary Information

**Additional file 1.** **1. Definitions. Table S1:** NEWS; **Table S2:** SOFA score; **Table S3:** CURB-65 scoring system; **Table S4:** PSI. **2. ARDS data. Table S5:** Characteristics and clinical parameters of ARDS patients.

## Data Availability

The data from this study are available from the corresponding author upon request.

## Abbreviations

APACHE II: Acute physiology and chronic health evaluation II
ARDS: Acute respiratory distress syndrome
CURB-65: Confusion, urea nitrogen, respiratory rate, blood pressure, 65 years of age and older
FiO_2_: Fraction of inspired oxygen
IFA: Indirect fluorescent antibody
LR: Likelihood ratio
MPR: Mean platelet count volume to platelet count ratio
MPV: Mean platelet volume
NEWS: National early warning score
PaO_2_: Partial pressure of arterial oxygen
PCR: Polymerase chain reaction
PMN: Polymorphonuclear leukocytes
PSI: Pneumonia severity index
SOFA: Sequential organ failure assessment
WBC: White blood cells
