# Supplementary material for: Predicting acute respiratory distress syndrome in influenza pneumonia patients using delta mean platelet volume
Source: BMC Pulm Med. 2021 Dec 7;21:405. doi: 10.1186/s12890-021-01763-5 (PMC8649995; doi:10.1186/s12890-021-01763-5)
Supplement: Supplementary file 1 — Additional file 1. 1. Definitions. Table S1: NEWS; Table S2: SOFA score; Table S3: CURB-65 scoring system; Table S4: PSI. 2. ARDS data. Table S5: Characteristics and clinical parameters of ARDS patients. [file 12890_2021_1763_MOESM1_ESM.docx]

**Supplementary data**

**Supplementary data 1: Definitions**

**Table S1** National early warning score (NEWS)

| **Scores**  **Parameters** | **3** | **2** | **1** | **0** | **1** | **2** | **3** |
| --- | --- | --- | --- | --- | --- | --- | --- |
| Respiratory rate (/min) | ≤ 8 |  | 9–11 | 12–20 |  | 21–24 | ≥ 25 |
| Heart rate (/min) | ≤40 |  | 41–50 | 51–90 | 91–110 | 111–130 | ≥131 |
| Systolic blood pressure (mmHg) | ≤90 | 91-100 | 101–110 | 111–219 |  |  | ≥220 |
| Temperature (°C) | ≤35 |  | 35.1–36 | 36.1–38 | 38.1–39 | ≥39.1 |  |
| Oxygen saturation (%) | ≤91 | 92–93 | 94–95 | ≥96 |  |  |  |
| Any supplement oxygen |  | Yes |  | No |  |  |  |
| Level of consciousness |  |  | Responds  to voice | Alert |  |  | Responds to voice, pain or unresponsive |

**Table S2** Sequential Organ Failure Assessment (SOFA) score

| **Score**  **Parameter** | **0** | **1** | **2** | **3** | **4** |
| --- | --- | --- | --- | --- | --- |
| Cardiovascular | MAP ≥ 70 mmHg | MAP < 70 mmHg | Dopamine <5 μg/kg/min  Dobutamine (any dose) | Dopamine 5.1–15 μg/kg/min,  Epinephrine or Norepinephrine  ≤ 0.1 μg/kg/min | *Dopamine > 15 μg/kg/min,  Epinephrine or Norepinephrine > 0.1 μg/kg/min |
| Respiratory  PaO_2_/FiO_2_ (mmHg) | ≥400 | <400 | < 300 | <200 with respiratory support | <100 with respiratory support |
| Renal  creatinine, (mg/dL) | <1.2 | 1.2–1.9 | 2.0–3.4 | 3.5–4.9 | >5.0 |
| Urine output (mL/day) |  |  |  | <500 | <200 |
| Liver,  bilirubin, (mg/dL) | <1.2 | 1.2–1.9 | 2.0–5.9 | 6.0–11.9 | >12.0 |
| Coagulation  platelets, ×10^3^/uL | ≥150 | <150 | <100 | <50 | <20 |
| Central nervous system/Glasgow Coma Score | 15 | 13–14 | 10–12 | 6–9 | <6 |

**Abbreviations:** MAP, mean arterial pressure; FiO_2_, fraction of inspired oxygen; PaO_2_, partial pressure of arterial oxygen

**Table S3** CURB-65 scoring system

| **Clinical feature** | **Points** |
| --- | --- |
| Confusion | 1 |
| Urea > 7 mmol/L | 1 |
| Respiratory rate ≥ 30 breaths/min | 1 |
| Systolic blood pressure ≤ 90 mm Hg or diastolic blood pressure ≤ 60 mm Hg | 1 |
| Age over 65 years | 1 |

**Table S4** Pneumonia severity index.

| **Factor** | **Score** |
| --- | --- |
| Patient age |  |
| Male | Age |
| Female | Age -10 |
| Long-term care facility resident | +10 |
| Accompanying disease |  |
| Neoplastic disease | +30 |
| Liver disease | +20 |
| Congestive heart failure | +10 |
| Cerebrovascular disease | +10 |
| Chronic kidney disease | +10 |
| Symptom at diagnosis |  |
| Acute psychosis | +20 |
| Breathing rate ≥ 30/min | +20 |
| Systolic pressure < 90 mm Hg | +15 |
| Body temperature < 35 °C or ≥ 40 °C | +15 |
| Heart rate ≥ 125/min | +10 |
| Laboratory measurements |  |
| Arterial blood pH < 7.73 | +30 |
| Blood urea nitrogen ≥ 30 mg/dL | +20 |
| Serum sodium < 130 mEq/L | +20 |
| Serum glucose ≥ 250 mg/dL | +10 |
| Haemoglobin < 9 mg/dL | +10 |
| Partial pressure of oxygen < 60 mm Hg | +10 |
| Pleural effusion | +10 |

**Supplementary data 2: ARDS data**

**Table S5** Characteristics and clinical parameters of ARDS patients (n = 56)

| **Characteristics** | **Value** |
| --- | --- |
| Days from hospital admission to develop ARDS | 3 (1–6) |
| Initial mechanical ventilation setting |  |
| TV (mL/kg of PBW) | 10 (9–12) |
| PEEP (cmH_2_O) | 5 (5–6) |
| FiO_2_ (%) | 40 (40–60) |
| Initial PaO_2_/FiO_2_ for diagnosis of ARDS | 192.5 (142–250) |

**Note:** Data are presented as median (interquartile range)

**Abbreviations:** ARDS, acute respiratory distress syndrome; FiO_2_, fraction of inspired oxygen; TV, tidal volume; PaO_2_, partial pressure of arterial oxygen; PBW, predicted body weight; PEEP, positive end-expiratory pressure
